# Perspective: The promise of multi-cellular engineered living systems

**DOI:** 10.1063/1.5038337

**Published:** 2018-10-11

**Authors:** Roger D. Kamm, Rashid Bashir, Natasha Arora, Roy D. Dar, Martha U. Gillette, Linda G. Griffith, Melissa L. Kemp, Kathy Kinlaw, Michael Levin, Adam C. Martin, Todd C. McDevitt, Robert M. Nerem, Mark J. Powers, Taher A. Saif, James Sharpe, Shuichi Takayama, Shoji Takeuchi, Ron Weiss, Kaiming Ye, Hannah G. Yevick, Muhammad H. Zaman

**Affiliations:** 1 Massachusetts Institute of Technology, Boston, Massachusetts 02139, USA; 2 University of Illinois at Urbana-Champaign, Urbana, Illinois 61820, USA; 3 Georgia Institute of Technology, Atlanta, Georgia 30332, USA; 4 Emory University, Atlanta, Georgia 30322, USA; 5 Tufts University, Boston, Massachusetts 02153, USA; 6 Gladstone Institutes, San Francisco, California 94158, USA; 7 Thermo Fisher Scientific, Frederick, Maryland 21704, USA; 8 EMBL Barcelona, European Molecular Biology Laboratory, Barcelona 08003, Spain; 9 The University of Tokyo, Tokyo 113-8654, Japan; 10 Binghamton University, Binghamton, New York 13902, USA; 11 Boston University, Boston, Massachusetts 02215, USA

## Abstract

Recent technological breakthroughs in our ability to derive and differentiate induced pluripotent stem cells, organoid biology, organ-on-chip assays, and 3-D bioprinting have all contributed to a heightened interest in the design, assembly, and manufacture of living systems with a broad range of potential uses. This white paper summarizes the state of the emerging field of “multi-cellular engineered living systems,” which are composed of interacting cell populations. Recent accomplishments are described, focusing on current and potential applications, as well as barriers to future advances, and the outlook for longer term benefits and potential ethical issues that need to be considered.

## I. INTRODUCTION

Over the past several decades, bioengineers, biophysicists, and biologists have made steady progress toward the creation of “multi-cellular engineered living systems” (M-CELS). These systems are composed of living cells and tissues organized in a way that produces novel functionalities by design. For present purposes, we consider the subset of M-CELS composed of mammalian cells and used primarily for biomedical applications and exclude, for example, other potentially important applications such as those in plant systems, energy harvesting, or the microbiome. Defined in this way, it includes organ-on-chip or tissue chip systems being developed for drug screening or disease models^1,2^ with the potential to expedite drug discovery and provide important new insights into fundamental disease processes. It also encompasses implantable “hyper-organs,” ones that, for example, sense a biological signal and synthesize and secrete a biologic product in response. Also included are biological actuators or bio-robots that have applications in various fields. These M-CELS might be assembled *in vitro* from clusters of individually differentiated cells or co-differentiated within a single aggregate of pluripotent cells. An important distinguishing feature is that these systems are designed to possess a specific form and function by design to perform in ways that are not found in natural systems today and ultimately that they can be produced in quantity and in a sufficiently robust manner, thereby making them reliable and amenable to large-scale manufacture.

While we have a tremendous knowledge base to draw upon for the design and manufacture of M-CELS, derived from the study and design of non-biological engineered systems, much is not directly applicable to M-CELS. This is a consequence of at least two important features that distinguish M-CELS from abiotic systems: first, our lack of a fundamental understanding of their inherent complexity, and second, the central role played by emergence in M-CELS formation. In this context, we define emergence as a self-directed, multicellular response occurring as a result of collective interactions of individual cells between themselves and the extracellular environment at microscale which manifests itself by phenomena at macroscopic, system-level scale. Living systems, even at the level of a single cell, are remarkably complex. Cells employ a vast array of signaling pathways to govern their phenotype and behavior, and when used as the building blocks of multi-cellular systems, the complexity quickly becomes overwhelming. Notably, models that are capable of predicting the phenotype of even a single simple cell from its genotype are only now becoming available.^3^ When multiple cells and cell types interact, new phenomena and properties emerge which can only be attributable to their collective behavior and extend far beyond the capabilities of single cells. While these collective, emergent behaviors are in principle predictable, they are enormously complex and arise from biological reactions that are only partly understood. While there is little doubt that the transition from single-celled organisms to more complex multicellular ones was absolutely essential for the richness of form and function we see in living systems today, our ability to understand and predict cell population behaviors remains nascent.

In order to make meaningful progress in developing the methods and tools needed to create M-CELS, we must draw upon expertise from various disciplines. Certainly, various biological sub-disciplines—synthetic biology, developmental biology, systems biology, and stem cell biology—are essential as are engineering approaches reflected in biomaterials and tissue engineering. However, we must also look into basic engineering design and manufacturing and a variety of enabling technologies, in order to make meaningful progress. Relevant to this, the need for “convergence” was recognized and articulated in the NRC Report, “Convergence: Facilitating Transdisciplinary Integration of Life Sciences, Physical Sciences, Engineering and Beyond.”^4^ Convergence remains key to the development of M-CELS, across numerous sub-fields (Table I).

**TABLE I.** Disciplines needed for progress in M-CELS and brief description of their respective contributions.

Discipline or sub-discipline	Contributions to M-CELS
Developmental biology	Understanding emergence, morphogenesis, and repair of complex morphologies in multi-cellular systems
Stem cell biology	Providing the source cells for M-CELS
Synthetic biology	Engineering robust genetic regulatory networks for co-differentiation and gene editing to control cell behavior and regulate time-dependent protein synthesis
Mechanobiology	Understanding how to control mechanical stimuli in a spatiotemporal manner in order to direct cell and tissue behavior and regulate co-differentiation
Tissue engineering	Creating the ability to design and fabricate simple multicellular constructs for medical applications
Biomaterials	Providing appropriate cell-matrix scaffolds and mechanical and chemical stimuli for M-CELS growth and stability
Biofabrication and manufacturing	Developing a new approach to manufacture M-CELS that accounts for emergence and complexity not present in abiotic systems
Multi-scale computational modeling	Creating predictive platforms for the design of M-CELS for specific functions
Ethics for M-CELS	Facilitating an open dialog on the benefits and potential concerns in M-CELS, generating a “code of ethics” to guide researchers

While steady progress in each of these disciplines fuels our ability to create M-CELS, several major recent advances are particularly enabling and noteworthy. First, the ability to reprogram adult, committed cells to generate induced pluripotent stem (iPS) cells^5^ has freed us from reliance on embryonic stem cells to form new organs or other systems. We can now differentiate induced human pluripotent stem cells (iPSCs) into a variety of cell types and begin to construct complex systems with a consistent genotype. Second, recent success in the growth of organ-like structures—organoids—from embryonic stem (ES) or iPS cells has shown that it is possible to co-differentiate cells into multiple cell types that begin to show the form, and in some cases, the function, of a real organ.^6^ Third, with the help from several new government programs worldwide, these technologies have been brought to bear on the growing field of tissue- or organ-on-a-chip development.

**FIG. 1.**
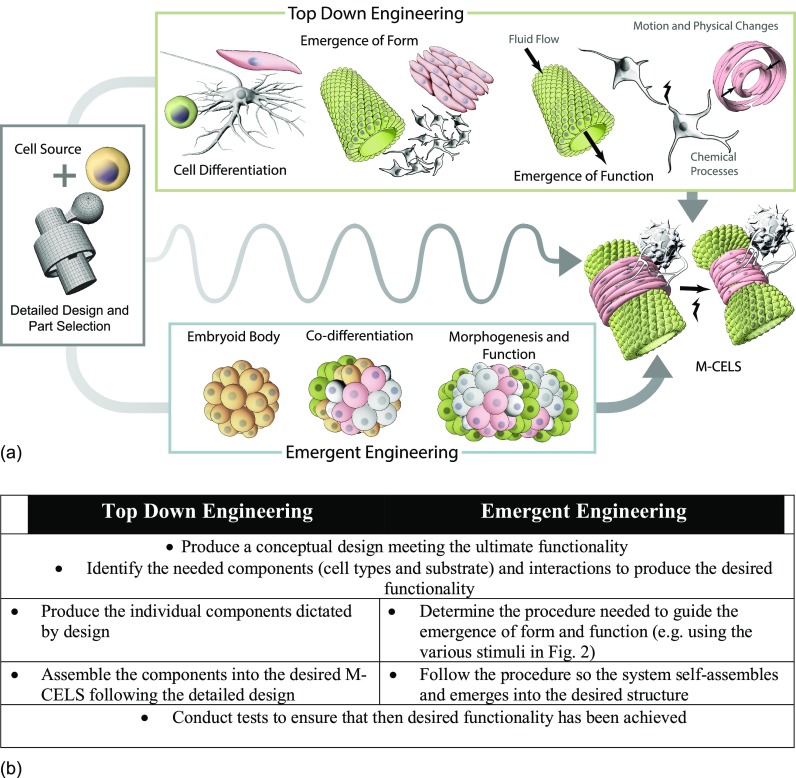
(a) Pathways to building a M-CELS. (b) Key steps to achieve the two distinct pathways. The wavy line in (a) shows that integration across the two pathways could also be possible.

As we develop the enabling technologies for M-CELS, at least two approaches for fabricating M-CELS can be envisioned (Fig. 1), here viewed in the context of a simple muscle-actuated sphincter or pump that requires a vessel lumen for flow, muscle to contract or collapse the vessel locally, and neural control, possibly by the use of optogenetically modified cells and activation by light. Both approaches begin with a concept and detailed mapping of the various cell types needed to produce the end-product. In one approach, differentiated pluripotent or primary cells are seeded or plated in a specified spatial pattern in two or three-dimensional constructs to produce the elements of the system. These may include, for example, an endothelial monolayer, a pancreatic islet, a muscle strip, or a cluster of neurons. These elements are then arranged within a device or substrate in such a way that they interact with each other in a desired manner. We liken this approach to current “top-down” engineering design and assembly of complex systems from simpler components, all according to a master plan for the complete, assembled M-CELS. In contrast, one could fabricate a M-CELS beginning with a disordered collection of pluripotent cells that are subjected to a variety of guidance cues—chemical, mechanical, electrical, and genetic—either globally or locally, which induce the cells causing them to co-differentiate into multiple cell types and self-organize into a new multi-cellular system. We define this to be an “emergent engineering” approach. Figure 1 shows these two pathways and the potential for interactions or cross-over between them.

At this early stage in the development of M-CELS technologies, we recognize the need to look ahead and consider the eventual scientific, commercial, and ethical impacts of our approaches and actions. The purpose of this paper is to provide a review of recent accomplishments, assess current state-of-the-art in M-CELS, and point to future needs and challenges. We also seek to foster a wider conversation of these issues, initiated in a *Workshop on Engineered Living Systems*, held in Chicago, IL, in August 2016 and continued in August 2018.

## II. DEVELOPMENTAL PROCESSES AND REGENERATION

The intricacy of the tissue/organ self-assembly represents the essence of what engineers strive to emulate as we design M-CELS. In organoid systems, we seek to replicate, control, and even deviate from normal developmental processes, so it is key that we understand these at a fundamental level. In addition to the initial emergence of form via the progression of genomic and biophysical processes, development and regeneration offer numerous examples of self-organization and dynamic pattern homeostasis even after initial anatomical organization is achieved.^7^

The correct stereotypical size and morphology of a developing organ result from the interplay between signals that regulate individual cell behavior and mechanical and biochemical feedback that emerges from interactions among many cells.^8–12^ A well-studied example of how the organ size is achieved via this interplay is the *Drosophila* imaginal wing disc, which over a period of 5 days grows from ∼50 to ∼50 000 cells and then stops. This growth is guided by both the spatially distributed expression of growth factors (e.g., *Drosophila* bone morphogenic protein (BMP) and Wnt homologues)^13^ and their resulting gradients and by mechanical forces which influence tissue growth.^14,15^ A model that has emerged from this system is that the organ size reflects an intricate balance between biochemical signals and tissue mechanics.^16,17^

Many kinds of embryos, especially mammals, can be split in half or joined together, still resulting in a perfectly normal animal,^18,19^ revealing regulative pattern control. After embryonic development, tissues can undergo various degrees of regeneration, supported by stem cells and their niches. One example is the mammalian intestine, where in humans, the small intestinal epithelial lining is renewed every 5 days. Other animals, such as salamanders, can regenerate whole limbs, spinal cords, jaws, eyes, hearts, and portions of their brain, after damage.^20,21^ An important aspect of regeneration is that, similar to embryonic growth, regeneration stops when a structure of the correct size and shape has formed.^22^ A better understanding of how the correct dimension feeds back to alter cell differentiation, migration, and proliferation in precisely the right way to bring the pattern closer to the appropriate target morphology would allow for an altered homeostatic size and tissue function.^23^ Taken together, exploiting innate pattern-restoration mechanisms would allow M-CELS to adapt to external perturbations and recover rapidly from damage. This could be applied, for example, to M-CELS that can respond to atrophy in a native tissue, growing and maintaining its complementary size.

In addition to set-points for the organ size, there is organismal-level homeostatic control. Planarian flatworms shrink and grow in allometric proportion, remodeling their entire body continuously to the available cell number due to eating or starvation.^24^ Tails transplanted onto the flanks of salamanders slowly remodel into limbs—a structure more appropriate to their new location.^25^ Additionally, tadpoles with experimentally rearranged Picasso-style faces still metamorphose into normal frogs, as individual organs migrate through non-endogenous paths as needed to build the correct frog face.^26^ The ability of an engineered organism to maintain its correct shape through similar mechanisms is another strategy to ensure robustness upon damage either during or after initial assembly, representing an important design challenge for a synthetic morphology.

A key issue for the future of M-CELS and regenerative medicine is to find the appropriate paradigm with which to enable direct modification of the shape for engineering and biomedical applications. Currently, the assembly of correctly shaped and patterned tissues is limited by our ability to manage their construction at a micro-level (e.g., cell type, cell position, and scaffold used)—a major barrier to progress in the repair of complex organs such as hands and eyes. These constructs are difficult to assemble directly but are routinely regenerated *in vivo* by some model species. Alternatively, the self-assembly of organoids leverages endogenous patterning cascades but comes at the cost of losing control over the final shape and pattern of the M-CELS. Ultimately, a solution lies between these extremes, as a kind of guided self-assembly that provides judicious tweaks to an otherwise self-directed endogenous cascade of morphogenesis,^27,28^ as further discussed in Sec. III. Advances in the field will depend on finding the minimal amount of input required for a system to progress towards a desired complex outcome. Promising approaches include activating master regulators of developmental programs (e.g., “build an eye here”) and learning to modulate biochemical and mechanical signals that trigger pattern formation above the cell level (e.g., organ size and spatial relationship between organs). Ever finer-scale reductionist analyses of the mechanisms of signaling at the cellular and sub-cellular levels, which are progressing at a very rapid pace, are complemented by theoretical and experimental approaches that quantitatively investigate the top-down, computational aspects of pattern regulation employed in tissue shape generation and maintenance.^29–31^

It is essential to understand and exploit the inherent processes that direct patterning of complex structures.^32^ Cells and tissues need to make many decisions about what to build, where, and when, in the control of growth and form. In our example of a muscle-actuated pump of Fig. 1, multiple cell types arranged in a specific spatial pattern are needed. The required structure bears similarities to the embryonic heart that develops in a remarkably robust manner in vertebrates,^33^ so we know that such signals can, in principle, be created and potentially deciphered. The logic of such processes is beginning to be understood, as inferred from studies of molecular regulatory networks,^34^ the encoding of positional information,^35^ and bioelectrical encoding of target states.^36^ Together, the biochemical and biophysical layers are being investigated with the tools of information theory, control theory, and computational cognitive science to develop interventions that target the biological systems' perception, memory, and decision-making about large-scale (morphological) properties.^28,37^ Moving beyond regulating just a few molecular pathways, we will be able to provide multiplexed inputs to tissues which alter the perception space of cells, leading to activation of desired morphogenetic and repair responses. It is becoming increasingly possible to leverage the ability of tissues to robustly implement specific modular patterning tasks via self-modeling and goal-directed remodeling activity.^38–41^

## III. CONTROLLING EMERGENCE

**FIG. 2.**
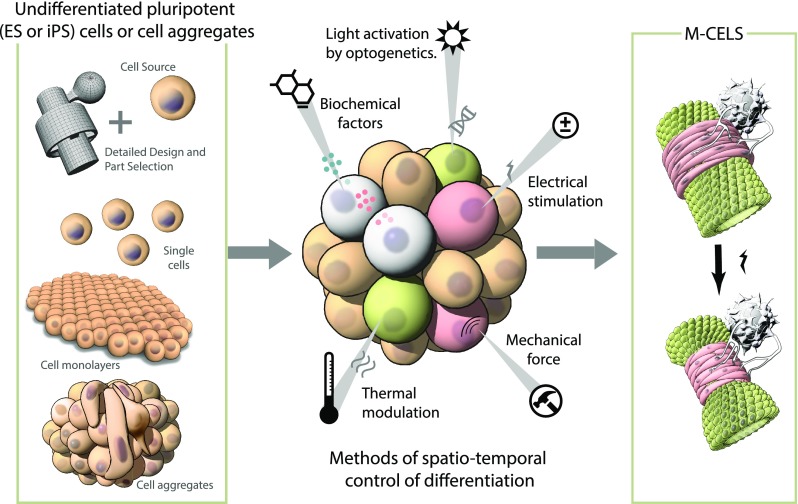
Methods for controlling emergence in M-CELS. Following the design specifications (left), a collection of procedures can be applied in a spatiotemporal manner to induce differentiation and organization of the cells (middle) to produce the desired form and function (right).

One of the greatest challenges in creating M-CELS is to regulate the spatio-temporal differentiation of cells within a developing construct such as an organoid. This control can be exerted in a variety of ways (Fig. 2). It has long been known that transcription factors, growth factors, morphogens, etc., play a key role in cell-cell or cell-matrix communication, and these exert their influence through activation of a multitude of intracellular signaling pathways. As mentioned in Sec. II, morphogens and their spatial gradients control many aspects of early development and determine much of the pattern formation that occurs as cells respond in diverse ways to various, and often multiple, local morphogen concentrations. In the case of organoid systems, morphogens are often used to push cell differentiation down to a specific path in order to produce a certain cell type of the desired tissue. In the muscle-actuated sphincter or pump, we require endothelial cells for the vessel, skeletal muscle for the pumping action, and motor neurons for controlled activation. However, achieving the needed level of control is clearly beyond current capabilities, as it is a major challenge to manipulate concentration gradients with the precision found in embryos. So, this method lacks the spatial specificity required to produce tissues with multiple cell types by design, as needed in the generation of a complex M-CELS such as the pump of Fig. 1.

In the same way that chemical factors act to control the biochemical signaling pathways, cellular and extra-cellular mechanics can also exert control over the cell function during development.^42^ Force transmission through matrix, cells, and their constituent molecules, in fact, can be thought of as a parallel “mechanical signaling pathway,” one that communicates with the biochemical pathway via several channels, including changes in molecular confirmation. Thus, as a result of force transmission through the extracellular matrix (ECM), cell-cell junctions, the cytoskeleton, or the membrane, proteins might alter their binding affinities or expose cryptic binding sites, stretch-activated ion channels might open, and cell surface receptors might become activated.^43,44^ However, just as in the case of biochemical control, mechanical control remains a relatively blunt tool to use for co-differentiation of cells into a desired spatial pattern. Methods are being developed, however, drawing upon existing technologies such as optical or magnetic tweezers or AFM, which offer finer control. Just as cells secrete factors to control biochemical signaling, cells can also exert mechanical force internally or externally, as a means of regulating their interactions.

An important layer of the morphogenetic field that orchestrates cell behavior is electric. All cells, and not just excitable nerve and muscle cells, communicate electrically using the same ion channels, neurotransmitters, and electrical synapses (gap junctions) which evolution optimized into brains. Parameters such as resting membrane potential (V_mem_) control proliferation, migration, cell shape, and differentiation^45,46^ and interact with chemical gradients, gene-regulatory networks, and tensile forces. More importantly, recent studies revealed that the dynamics of bioelectric networks process a kind of tissue memory that specifies high-level properties such as size control, organ identity, and axial polarity of the entire body plan.^47^ Recent developments of pharmacological and optogenetic techniques for specific manipulation of bioelectric circuits in non-neural, somatic tissues have shown the ability to trigger appendage regeneration, reprogram gut into complete eyes, alter the number of legs and heads, reprogram tumors into normal tissues, and overcome mutations in key patterning genes.^48,49^ Bioelectric properties control gene expression and chromatin state via a number of known transduction mechanisms; major opportunities for advances in this field focus on cracking the bioelectric code—the mapping of large-scale voltage gradient patterns onto organ-level outcomes.^50,51^ Given the degree of plasticity revealed by bioelectric controls of growth and form, advances in this field will be an invaluable aid in the top-down programming of the shape in bioengineering applications.

Genetic engineering of cells allows us “from the inside” to modify and extend the biological programs that underlie cellular behavior, through the design and implementation of synthetic gene regulatory networks.^52^ With the advent of the Cas9/CRISPR system,^53^ it is now possible to edit the genome and dynamically regulate specific genes. Synthetic biology has evolved from demonstrating a simple gene circuit in prokaryotes^54,55^ to large multi-input circuits in prokaryotes^56^ and eukaryotes.^57^ These circuits usually comprise genetically encoded sensors that detect levels of intracellular and extracellular biomarkers, a computational core that processes sensory information and makes decisions about which specific actions to take, and actuators that affect the cell state and the surrounding environment. Initial emphasis in synthetic biology focused on the creation of gene circuits that operate orthogonally to the cell, attempting to minimize bidirectional interactions and dependencies between the circuit and the host. In contrast, genetic circuits fashioned for M-CELS must at their core be conceptually and practically embedded within the host and tissue, responding to dynamically changing cellular and extracellular conditions and controlling the cellular milieu towards desired phenotypes.

Unlike many traditional approaches in engineering, M-CELS must develop the capability to cope with or exploit the heterogeneity of cell types, states, environmental conditions, and fluctuations in gene expression (or “noise”). Engineering noise of M-CELS will require addressing noise at multiple-scales throughout development. Predictive modeling and advanced tools to modulate the noise of genetic and regulatory circuits within the cell will be needed.^58–61^ Addressing these issues within an ensemble includes accounting for the coupling of fluctuations in cell-to-cell signaling and the microenvironment. This presents an opportunity to develop noise engineering approaches in both single-cell and multi-cellular systems. Engineering stochasticity using synthetic systems^58,59^ or exogenous drug targeting^60^ of gene circuits and regulatory motifs and networks can bias cell-fates and provide a role for stochastic noise engineering of growth and pattern development.^62,63^ Integration of engineering noise and stochasticity into the fundamental framework of bioengineering living systems can account for an iterative design of fluctuations in signaling pathways at both single- and multi-cellular levels.

Other methods that hold promise for localized control in M-CELS are optogenetics,^64^ using light-activated ion channels, and magnetogenetics,^65^ locally activating ion channels, applying force, or precisely controlled heating. In these, either light or magnetic fields, often in combination with nanoparticles, are used to control local processes. For example, in the sphincter/pump of Fig. 2, activating spatially separated cells to differentiate into the three indicated cell types might be accomplished by localized stimulation. This might be accomplished by focusing light of different wavelengths at cells engineered to synthesize specific transcription factors through the use of optogenetics. The capability of such spatiotemporal activation, however, has yet to be demonstrated, and remains an obstacle to progress.

Thus, cells possess a variety of regulatory mechanisms, and each of these provides a means of regulating cell differentiation and function in the generation of M-CELS and, in particular, to exert control over the morphogenesis of organoid systems. To do so requires more than merely a collection of “tools” that can be used to manipulate the system; instead, it would need a systematic approach to gaining a more fundamental understanding of the principles that determine form and function. Moreover, such an understanding would then facilitate generation of a set of design principles, as described in Sec. VII. This represents one of the major challenges of the field going forward.

## IV. ORGANOIDS

Several recent scientific advances have helped seed the nascent field of M-CELS, but none more so than those in organoid culture systems. Organoid, in this context, refers to an aggregate of multiple organ-specific cell types structured similarly to the *in vivo* organ and sharing its key functions. Research in organoids, built on work that started in the early 1900s,^66^ is now accelerating as it incorporates recent advances in stem cell biology. Numerous publications have identified culture conditions that support the differentiation of pluripotent stem cells (PSCs) and the differentiation and/or self-renewal of adult stem cells in a manner that produces organoids.^67,68^ PSCs have been differentiated into organoids representing a variety of organs or complex systems including but not limited to the liver, brain, small intestine, stomach, pancreas, lung, kidney, and esophagus.^69–80^

While there are large gaps in our understanding of the mechanisms of organoid formation, such as the rules that govern morphogenesis, regulate the size and shape, and lead to the emergence of adult stem and progenitor populations, researchers are able to successfully maintain organoids in culture for extended periods of time.^67,77^ The most common approach to developing an organoid culture system has been to follow nature's template, translating findings from developmental biology to direct differentiation *in vitro*. However, there are likely alternative paths that could produce similar results because *in vitro* systems are freed from evolutionary pressures and the dynamics of a complex organism. Discovering these “short circuits” to achieve emergent organoid features will likely be a vibrant area of research moving forward. A point of caution is whether or not the resulting organoid from such an approach will be sufficiently similar in form and function to the organ and whether or not it will suffice for its engineered purpose.^81,82^

Despite the advances made in the last six years in a variety of tissue types, many challenges remain to further development of organoid systems. Many of these challenges stem from a lack of fundamental understanding associated with the biochemical, physical, and mechanical drivers of differentiation within a multicellular environment which give rise to aggregates with the form and function of organs. For example, how do asymmetrical structures emerge? What structural components are critical for the emergence of the desired function? And why is there such variability in how PSCs respond to morphogenic cues? Some of the variability is due to stochasticity or underlying biological noise (see also Sec. III). Engineering approaches may ultimately leverage this stochasticity to drive differentiation or “canalization” in directions that otherwise would be unfavorable under naturally occurring conditions. The same features that give rise to cellular heterogeneity, however, also result in inconsistency with organoid differentiations regardless of the tissue type, which is a barrier to wider scale adoption of organoid systems in industrial screening settings.

Before organoids can readily be incorporated into M-CELS or used at an industrial scale, there are practical implementation issues to address. First, at each stage of organoid formation, the ability to scale in batches for suitable use in a high-throughput screen (e.g., >10 000 small molecule compounds) is limited. Second, existing protocols do not address a fundamental issue of how heterotypic tissues may have varied nutritional requirements and media needs; currently, a given organoid will be supported by a common medium and culture conditions. Third, organoids can be cultured long-term, but like other PSC derivatives, they tend to arrest developmentally before reaching a mature, adult-like stage.^83^ Furthermore, the current repertoire of organoids does not contain all organ-specific cell types or contain mature cell types from other systems, like the circulatory or nervous systems. Finally, most of these organoid systems still rely on undefined, highly variable components such as Matrigel and serum, which restricts our ability to control and fine-tune the emergence of organoids. These factors contribute to the variable rates of success in creating an organoid and our inability to alter the resulting structure, by design, to produce new functionalities.

Many groups are working to address these issues both by furthering our understanding of the principles governing organoid emergence and also developing new tools to aid in these studies and provide new methods of manipulating the differentiation cultures. For example, the development of on-chip culturing devices provides more sophistication in geometric arraying for imaging and recovery for later biochemical analysis. Tunable hydrogels will enable detailed analysis of the role of the matrix,^84^ and biosensors for metabolic studies will be valuable tools to find common medium and culture conditions.^85^ Microfluidic devices can be designed to support analysis methods such as smFISH and immunofluorescence.^86^ Novel tools for multicellular image digitization into network properties^87^ have allowed for extraction and quantification of emergence, but more computational methods need to be developed in order to identify and predict desired features of organoid development, especially if large industrial-scale manufacturing of these systems is needed for the next generation of drug development.

## V. ORGAN-ON-CHIP MODELS

Building on the success of microfluidic technologies and iPS and stem cell engineering and driven by the widely recognized need for transformative changes in the process of drug discovery and development, a variety of new assays have been advanced which enable certain aspects of the organ function to be replicated with *in vitro* models. While the “organ-on-chip” (OoC) technologies are still at a relatively early stage in development, nascent versions of cardiac muscle,^88,89^ liver,^1,90^ brain,^91–93^ lung,^94^ skin,^95^ placenta,^96^ and various other tissues have been reported (Table II). Similar systems have been created for the purpose of modeling and gaining new insights into fundamental disease processes such as cancer^97^ and Alzheimer's disease.^98^ Given the increasing availability and reduced cost of generating iPS cells, the prospect of developing patient specific assays to screen for optimal personalized therapies is on the horizon.

**TABLE II.** Example organ-on-chip designs of different physiological functional units. A wide range of organ mimics have been reported which recapitulate the basic functionality of that organ.

Tissue or organ system on-chip	Description	References
Lung alveolus	Alveolus with endothelial and epithelial monolayers. Time-varying vacuum in side chambers provides for transient strain.	Huh^94^
Liver	Functionally active hepatocyte tissues maintained in a perfusable multi-well format.	Domansky^1^
Heart	Cardiac muscle is seeded on a flexible cantilever so that the magnitude of contractile force can be measured.	Lind^88^
Microvascular	Vascular networks formed by a vasculogenesis-like process	Kim^99^
Placenta	Multilayered system for co-culture of human trophoblast cells and human fetal endothelial cells replicating *in vivo* spatial organization.	White^87^
Skin	Skin represented by an epidermal layer is integrated with a perfusable vascular channel for cosmetic testing.	Morris^82^
Blood-brain barrier	Blood-brain-barrier containing an endothelial layer, astrocytes, and pericytes including the measurement of permeability.	Booth^100^ and Campisi^101^
Neurovascular	An endothelial barrier with interacting astrocytes and neurons in separate gel regions.	Adriani^93^
Neuromuscular junction	Neurosphere formed from optogenetic motor neurons extending neurites to connect with skeletal muscle.	Uzel and Morimoto ^102,103^

Practical challenges include phenotypic instability, low throughput associated with system complexity, material-drug incompatibilities of commonly used device materials such as PDMS, and biomaterial inconsistencies and limitations. A fundamental question for OoC technology is, will we be able to create microscale constructs that adequately recapitulate the macroscopic organs? There is a danger, even if we construct OoC using human cells with sufficiently mature phenotypes, that the resulting system will have the physiology more reminiscent of a small rodent rather than the humans from whom the cells were originally derived. Two major scaling issues arise in OoC design and construction: (i) maintaining absolute values of physiological parameters and (ii) maintaining relative sizes between different types of cells, tissues, and organs.

One example of changes in absolute physiological parameters due to scaling involves cellular level metabolic rates.^104^ As the size of an animal decreases, the basal cellular metabolic rate increases. Importantly, this does not occur because of cell-intrinsic differences in the metabolic rate. Instead, the basal metabolic rate of cells is dictated by the rate of nutrients and oxygen delivery. Thus, in the typical, nutrient-, and oxygen-plentiful culture environments, human cells and even faithfully constructed minimal functional tissue units would metabolize at mouse-like rates rather than human-like rates. While there are potential solutions such as limiting oxygen availability,^105^ effective implementation in OoC systems is still a challenge.

Even if one were to achieve human-like physiological parameters for each organ module, another challenge arises when connecting multiple OoCs together with a common medium that might be circulated by our muscle-actuated pump example. If we shrink all linear dimensions proportionally, the multiple OoC system will not reflect human proportions in terms of function. For example, if a lung is isometrically miniaturized by a factor of 100, the surface area available for gas exchange falls nearly 10 000-fold. The same change in linear dimension for muscle would reduce oxygen consumption rates by a factor of 10^6^. Some potential solutions include “functional scaling” of organs where miniaturization factors would be assigned to different organs based on whether the organ function depends more on its surface area or its volume.^104,106^ While these and other potential solutions have been proposed,^107–109^ effective implementation and validation in OoC systems remain a significant challenge.

While the long-term goal may be the construction of an OoC that reproduces all aspects of human physiology, the near-term opportunity may be in fit-for-purpose OoCs that test for specific aspects of efficacy or toxicity. Such efforts will require close collaboration with target end-users to develop OoCs with “just enough complexity” to provide a valuable physiologic readout while also allowing for a robust and high throughput assay. The systems will also have to be extensively validated against existing gold-standard animal models using a large panel of reference chemicals. A helpful resource with various examples of validating non-animal technologies is The European Union Reference Laboratory for Alternative to Animal Testing (eurl-ecvam.jrc.ec.europa.eu). Finally, the continuing difficulties in predicting adverse immune responses and the emergence of a range of successful immunotherapies suggest a need and opportunity for OoCs that replicate tissue-level aspects of immunity.

## VI. BIOLOGICAL ROBOTICS

An important application of M-CELS could be in the development of biological robotics and actuation systems.^110^ Over the years, engineers have produced a variety of non-biological robots and machines with an immense impact on our lives and industrial production. However, they have fundamental limitations when compared to living systems, e.g., they cannot self-emerge, self-assemble, or self-heal. On the other hand, in nature, meso- and macro-scale functional organs and organisms (machines) emerge through complex interactions between individual cells and the extra cellular matrix. Organs, in turn, interact with one another through precise control algorithms that maintain homeostasis. However, the rules of the interaction between living biological components at various hierarchies and spatio-temporal scales remain elusive. With new methods of culturing and manipulating living cells, there is a growing interest in developing cell-based biological robots, many with engineered scaffolds. It is envisioned that such robots may have unprecedented capabilities, as they could reap the benefits of evolutionary pressures.

As an example, the development of biological actuators can form the building block of more complex emergent systems. Such engineered biological actuators consist of muscle cells (primary, cell lines or differentiated from stem cells or iPSCs), supporting cells such as fibroblasts, and an extracellular matrix (ECM) and may require a scaffold as a substrate. The cells interact with the ECM and remodel it (compact and align fibers of the ECM), approach, align^111^ and fuse with each other, undergo myogenesis, and emerge as muscle strips anchored to the scaffold. Unlike cells in other organoids, muscle cells interact over long distances using mechanical force. These complex interactions, both local and distant, involving diffusion and mechanical forces, result in an emergent muscle system that possibly maximizes force and motion and minimizes energy demand. Thus, the design of a muscle system involves solving an inverse engineering problem—given a prescribed form and function of the system, choose the appropriate scaffold and its biomaterial, ECM, and the muscle cell type and density.

Using the above principles, engineered muscle systems have been developed for *in vivo* applications such as cardiac tissue repair^112^ and *in vitro* applications such as pumping fluids at small scale^113^ and phenotypic assessment of neuromuscular disease.^114^ A variety of muscle systems have been explored to achieve multi-dimensional and complex motion and deformation from muscle contractions on 2D and 3D substrates.^115^ These engineered systems serve as a test bed for studying muscle force, their temporal dynamics, longevity, and overall performance in contrast to their *in vivo* counterparts, with the ultimate goal of providing muscle repair, analysis of muscle disease, and evaluation of drug efficacy. More interestingly, it is now possible to use human iPSCs to form patient specific self-organized muscle strips to develop disease models for prognosis, to interrogate disease mechanisms, and to study drug effects^116^ for individualized medicine. Recognizing that muscles are often activated by neurons for both voluntary and involuntary motions, recent work with M-CELS also involves muscles innervated by neurons, forming functional neuromuscular junctions.^102^

The force and deformation attributes of engineered muscle systems have recently been employed to generate locomotion of small structures. They include swimming in fluids mimicking flagella dynamics at small scale (low Reynolds number),^117^ coordinated flapping dynamics at larger scale (high Reynolds number),^118–120^ and walking using leg-like structures.^115,121–124^ These small robots move autonomously with cardiomyocytes as actuators, or they are stimulated to move by light or an electric field when optogenetic or regular skeletal muscle cells are used to actuate them. Through these elementary systems, we are learning to imitate different capabilities of natural organisms, including locomotion and active transport, which can lead to new applications.^125^ These include future *in vivo* applications in drug delivery and micro-surgery. However, major challenges need to be overcome, including the issues with biocompatibility, imaging and control of trajectories, viability, and their removal after the desired function. In the near term, they serve as test beds for studying emergent properties of biohybrid robots arising from clusters of heterotypic cells, ECM, and scaffolds. In the medium and long-term, such top down engineered biological robotics could consist of many more cell types to achieve specific functions and applications in the environment, energy, medicine, and others.

## VII. DESIGN PRINCIPLES

Success of engineering systems rests on the formulation of effective design principles that guide the creation of complex systems. Accordingly, achieving the M-CELS goals of creating complex synthetic multicellular structures with defined behaviors will require the establishment of experimentally verified rules and practices that guide the efficient and predictable formation of these systems. The key to success will be the integration of traditional engineering concepts, such as creating and characterizing reusable parts, establishing rules for the composition of such parts, appropriate layers of abstraction, and modular system design, along with design principles that take into account the unique properties and interactions of the biological substrate. In other engineering disciplines, one can achieve desired behavior by exerting fine-grain control over system components. In contrast, while precise spatiotemporal control over specific elements of a living system is becoming feasible, regulating all aspects at all times is not realistic. As such, a different approach must underlie our biological system design efforts.

However, while traditional engineering approaches may have limitations, if they are pursued with regard to the biological substrate, it is important to note that M-CELS efforts should be inspired by, but not limited to, natural designs. Design principles that have emerged from the evolution of biological systems are instructive regarding what works and what does not work in a biological context. However, evolution is incremental and produces intermediate designs that are both functional and competitive within their environmental context. In contrast, M-CELS and their designs are not constrained in the same manner. Both top-down and emergent engineered systems may favor configurations that are easier to understand, intentionally support and simplify future enhancements and modifications, and arrive at solutions that leapfrog existing designs.

Several fundamental properties of living systems introduce challenges to engineering design. Importantly, the high-level behavior of living systems emerges from the properties and interactions between their constituent low-level components.^126^ To design M-CELS effectively, we must consider the vast range of scales in which they operate (from the subcellular to the organismal) and the need to integrate different control modalities. In biology, as in other areas, the system function is tightly interwoven with the structure. The structure/function relationship manifests itself at different scales of living systems, and it is also impacted by the numerous ways in which cellular systems exert control over themselves and their surroundings. In the efforts to engineer living systems, we have at our disposal a large set of proven methods for manipulating biological behavior. It can be modulated “from the outside” via micro and macroscale methods described in Sec. III.

To fully realize the potential of M-CELS, these control methods can serve as composable functions. These functions should have inputs and outputs and perform some transformation on the living systems. The functions operate in space and time and at defined scales. While they always perform operations physically, their impact can be both physical and regulatory/information-processing. Functions have specific energy usage, operate at different modalities, and may require coordination among multiple cells and time scales. While the concept of a function is relatively straightforward, implementing a design framework that effectively integrates different modes of control modalities as discussed in Sec. III remains a challenge. 3D multicellular simulation tools such as Morpheus^127^ and others^87,128^ integrate gene regulation, cell signaling, and biomechanics and may serve as a useful basis for M-CELS design tools. However, missing are effective abstractions that support M-CELS 3D organ and multicellular machine design objectives, akin to gene circuit design tools.^129–132^

## VIII. ENABLING TECHNOLOGIES AND COMPUTATIONAL METHODS

New scientific directions inevitably require new technologies. In the context of M-CELS, we have already encountered technological limitations for sensing, modeling, and reengineering of the form and function of cell-based systems. Technologies are needed for the formation and characterization of organoids and cellular clusters, 4D imaging of these biological systems, 3D dimensional patterning and fabrication, flexible means of providing bioelectric and metabolic stimuli during guided self-assembly, and computational approaches capable of predicting the behavior of cell populations of multiple cell types whose form and function emerge over time.

Enabling technologies can be categorized in terms of where they fit within the life cycle of a M-CELS. First, the systems need to be assembled or arranged into a configuration that facilitates emergence and self-assembly. Technologies such as 3D printing, biomaterial scaffold design, and advanced microfluidics have already demonstrated their benefits and potential but need further development. Microfluidics has the capability of culturing cells in 3D with multiple communicating cell types that can be arranged through 3D printing so as to facilitate the essential interactions needed to generate the necessary cell-cell and cell-matrix interactions and realization of autonomous operation of these systems in a wide range of environments and ambient conditions.

Methods are also needed to interrogate the developing systems and to assess their final outcome—the end product of the biomanufacturing process. Imaging will be key, but we are now facing limitations in terms of our ability to visualize the structures deep inside a M-CELS, especially in a label-free manner in live cells over time. Methods such as CLARITY^133^ and 3DISCO^134^ and their variations have already proven useful for imaging large multicellular constructs, but these, too, have limitations and provide little opportunity to assess the cell functionality of internal structures.

Another consideration is the need to assess system stability over time. Unlike abiotic systems, M-CELS have the tendency to continue to change with time. Some aspects of change—adaptability and self-repair—have benefits, but others such as loss of differentiation and progressive cell death are clearly detrimental to function. It will therefore be necessary to incorporate methods to continually assess the stability of the system so as to ensure its performance. Functional measures such as the secretion rate of an essential chemical or force generation are examples.

In addition to experimental work, computational approaches will be required at every step and closely coupled with experimental data, to analyze, predict, and probe the system's operation. Synthetic biology at the single-cell level has had many successes,^56,57^ but it has also had to accept that progress has been slower than once hoped.^135^ One of the many challenges is the non-intuitive behavior of molecular circuits: complex topologies, non-linear relationships, and feedback loops often make the dynamics of a circuit impossible to predict without the aid of computer simulations—these unexpected behaviors may be considered as examples of the *emergence*, discussed above. Much of this work still revolves around trying to understand the dynamics of simple circuits that are already constructed rather than trying to design *de novo* circuits, but this ambitious challenge of engineering new circuits is also gaining ground.^34,130–132^

Switching from single-celled circuits to multicellular systems brings a level of complexity for computer modeling. Feedback loops now exist at more scales than the purely molecular. Although the active dynamics of cells are known to be largely controlled by their molecular states, it is now equally clear that macroscopic events directly feed back to control molecular events. For example, tissue growth may push a group of morphogen-secreting cells away from their target cells, thus changing their expression profiles as a direct consequence. If the behavior of feedback loops in single-celled gene circuits is hard to predict, the complexity of these multi-cellular and multi-scale feedbacks is dramatically more challenging. That is, the degree of complex and subtle *emergence* is even higher than for molecular circuits alone. Understanding and predicting them will not be possible without good computer models, so a clear expectation for the future is that multi-scale numerical simulations must become a key goal for M-CELS.

A wide variety of modeling formalisms exist to tackle multi-cellular systems. At one end of the spectrum are continuum approaches such as Finite Element Modeling (FEM), which approximate a tissue as a continuous material, and they are optimal for questions involving physical mechanics.^136^ At the other end of the spectrum, Cellular Potts Models (CPMs) employ many discrete variables on a lattice of points to represent each cell of the tissue.^137^ They can therefore capture the irregular shapes of individual cells and have been used to simulate various developmental processes,^138^ as well as tumor growth and vasculogenesis.^139^ However, for general-purpose modeling of engineered tissues, their computational cost and limitations in representing the mechanical integrity of large-scale structures (e.g., macroscopic bone or cartilage elements) make them unlikely to become the primary formalism of choice for M-CELS. In between these two extremes are a collection of techniques which together may be termed off-lattice methods: *agent-based* models are often defined in terms of logic-based rules, or “state-charts,” and have been particularly successful for cases where the primary scientific question is about the control of differentiation through discrete cell states or fates.^140^ *Vertex models* in contrast have been used mostly to explore the physical mechanics of cellular interactions—how different types of cell divisions affect the packing and cell shape.^141^ Since this formalism explicitly represents the boundaries of cells, it is only really optimal for epithelial tissues having a neat polygonal packing arrangement. For volumetric 3D tissues such as mesenchyme, *particle-based* or *cell-centroid* models are preferable. They cope well with arbitrary 3D arrangements of cells, without the computational overhead of maintaining mesh integrity. At the same time, they are ideal for simulating the molecular aspect of the system—cell autonomous reactions can be calculated on each particle, and diffusion can be well-approximated by exchange of molecular concentrations between adjacent cells. Impressive results have recently been demonstrated for early zebrafish development and gastrulation.^142,143^

Despite exciting improvements in these modeling approaches, we should be cautious about predicting the use of computer modeling as a tool for “rational” design of multicellular M-CELS. Thus far, the design principles found to be useful tend to exist at specific scales. For example, at the lowest scale of small molecular circuits, we may use the design concepts of Boolean logic or dynamical systems theory (attractors in phase portraits, etc.).^34,56^ At the intermediate scale of multi-cellular pattern formation, we can employ the unifying concepts of positional information or self-organized patterning.^144,145^ While at the scale of macroscopic tissues, interactions between active tissue growth and the material response properties of the tissue can also lead to design principles based on physical mechanics (the control of stress distributions, buckling, etc.).^146^ However, the interactions between design principles at different scales have rarely been explored.^147^ Since a key feature of engineered tissues and organs is their multi-scale nature and we hope to control dynamic macroscopic processes through “molecular programs,” it will probably take a significant phase of further basic research before computer modeling aids us in “forward” rational design of these systems. Indeed, for the foreseeable future, computer modeling research will probably focus on obtaining satisfactory models of existing multicellular systems, and this will largely depend on “reverse-engineering” from large quantitative datasets. Organoids may prove a particularly tractable system to work on in the short term, due to their both relative simplicity (compared to organs or embryos) and visual accessibility for digitizing with time-lapse imaging.

## IX. BIOMANUFACTURING

The biomanufacture of M-CELS poses significant challenges to existing bioprocesses.^148^ M-CELS present unique needs based on their inherent complexity and reliance on emergent behavior. The overarching need—developing effective methods to reliably and robustly produce M-CELS at a desirable scale—highlights a number of challenges and opportunities.

The development of M-CELS relies upon the integrated formation of cellular structures. Such structures can be achieved through either self- or guided-assembly of multiple cell types into functional units (see Sec. I). As we consider methods for manufacturing these systems, it will be important to understand whether such complexity can be reduced or segmented in a modular fashion to create M-CELS subunits, followed by assembly into desired M-CELS in a controllable or bio-foundry manner. It is expected that efforts to better understand the phenomena associated with emergence will help elucidate where such processes may be simplified or deconstructed and where they cannot. The goal of such efforts is to enable the creation of a “bio-assembly line” to achieve industrial scale production of desired M-CELS.

**FIG. 3.**
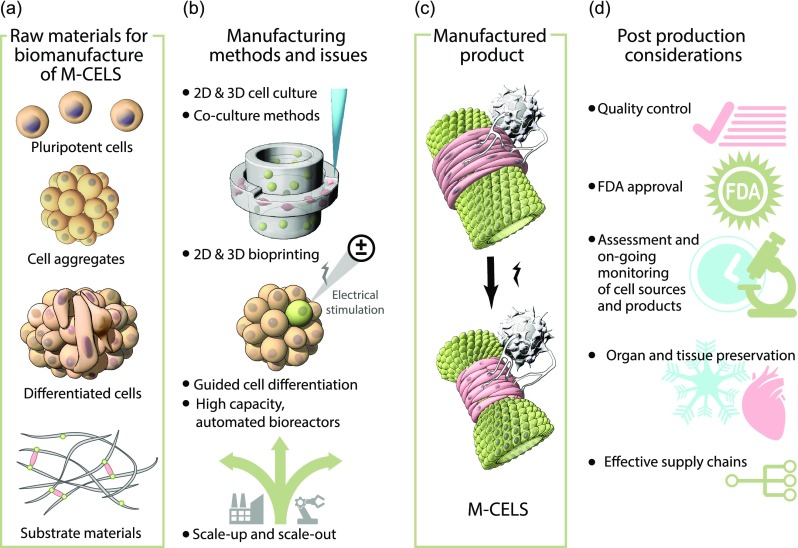
Manufacture of M-CELS. (a) Design and selection of cellular and substrate components. (b) Product manufacture requiring a variety of manufacturing methods, many being unique to biological systems. (c) The manufactured product. (d) Post-processing considerations.

Biofabrication of subunits and their further assembly into M-CELS requires manufacturing technologies across multiple disciplines (Table I) and technologies (Table III). Simple 2D and 3D cell culture methods can be augmented or replaced by newer technologies such as 3D bioprinting,^149–153^ and automated bioreactor systems are key to enabling the generation of compartmentalized M-CELS structures in an organized manner (Fig. 3). The continuing development of effective 3D bioprinting technologies may enable the effective implementation of this approach in M-CELS biomanufacture. Although the resolution that is currently achievable with 3D bioprinting may not yet permit direct implementation on the length scales (10's of microns) required for M-CELS formation, they can help, at least, spatially position cells in a manner that will facilitate further self-assembly and may prove to be effective tools for automating and standardizing key steps in M-CELS biomanufacturing.

**TABLE III.** New technologies needed for the development and manufacture of M-CELS.

Technology	Application to M-CELS
Imaging	High resolution, high content imaging of large, multi-cellular structures, label-free methods, and 4D imaging
Computational analysis	Multi-scale modeling, agent-based methods, and data-driven modeling
Bioprinting	Simultaneous printing of the matrix and multiple cell types with single cell resolution
Scaffold design	Artificial and natural biomaterials with controllable chemistry and mechanical stiffness
Microfluidics	Systems that facilitate spatiotemporal control of micro-environmental properties and co-differentiation processes
Biofabrication	Providing appropriate cell-matrix stimuli for organoid growth and stability and new manufacturing methods that leverage intrinsic self-assembly
Optogenetics	To facilitate the capability for spatiotemporal patterning of the function in growing M-CELS
Robotics	Methods to handle high-volume production of organoids and other M-CELS for industrial applications

Technologies that enable both scale up—relying on novel culture platforms with increased capacity (e.g., bioreactors) and scale out—increasing the number of culture systems in use—will be of paramount importance to M-CELS biomanufacturing (Fig. 3).^154^ Improved control of M-CELS biomanufacturing poses unique challenges based on the features that differentiate M-CELS from traditional cell based systems—cellular complexity and emergent behavior. Since cell-based processes are subject to inherent biological variability, the push towards more defined and controllable components for use as cellular matrices and media is imperative in minimizing “external” heterogeneity of M-CELS biomanufacturing. These variabilities highlight the challenge for quality control (QC). Traditional cell-based products such as primary cells or stem cells are developed with a mindset towards maximizing homogeneity of the cell type(s) associated with these systems. As we begin to tackle the challenges of M-CELS biomanufacturing, the heterogeneity of the cellular component is not just a possibility but a necessity. Methods to understand and control this heterogeneity are therefore essential in the successful biomanufacturing of M-CELS.

Furthermore, the biomanufacturing of M-CELS will necessitate the development of effective and appropriate assays for evaluating the M-CELS identity and function. These assays will include the characterization of cell sources and biomaterials using many of the technologies described in Sec. VIII. Ideally, these should be noninvasive or minimally-destructive to enable live and real time monitoring of developing M-CELS. As our ability to discern the mechanistic underpinnings of M-CELS development evolves, we also anticipate an increasing demand for the use of molecular and analytical analysis in the selection of input materials. We already see the consequences of the considerable variability in these fundamental components of a M-CELS—variability in organoid formation from the same starting population^155^ and disparity in directed differentiation of various pluripotent stem cells.^156,157^ These examples highlight differences that can occur under ostensibly identical extracellular environments. We anticipate that technologies such as targeted sequencing, micro RNA analysis, epigenetic fingerprinting, and proteomics will help elucidate and establish appropriate specifications for these cells, but much work is needed before adequate procedures can be put in place.

Finally, the biomanufacturing of M-CELS demands the development of reliable tissue preservation technologies. While laboratory-scale M-CELS can be made and used on-demand,^148^ industry-scale production of these systems includes release and distribution of M-CELS through supply-chains that are dependent upon reliable preservation. Cryo- or other preservation technologies of M-CELS pre-products or sub-assemblies will streamline and simplify the manufacturing process. The ability to preserve completed M-CELS could enable building “finished-goods” inventories and shipping to end-users.

## X. ETHICAL CONSIDERATIONS

A fundamental question for researchers working in synthetic biology, emergent behavior, and living systems is whether they are “creating life.” Some researchers in synthetic biology believe microorganisms are “just” machines, and that the creation of new such machines shouldn't be considered differently.^149^ Others may argue that researchers need to be cognizant of the degree to which projects might be perceived as creating life and what special obligations might exist in this creative process. The language of “creating life” raises fundamental questions, including religious issues, for some observers who ask whether there should be natural limits beyond which we should not be trespassing. While many of these issues have been raised in the context of synthetic biology, they grow in importance when, for example, we become able to produce a functioning brain organoid that can collect, process, and act based on information gained from the environment. In addition, the concept of pain and sentience^150,151^ would need to be addressed in the development of M-CELS that could be perceived as replication of living entities. As many of the future M-CELS could by pass the natural developmental process, the use of the 14 day rule^150,152^ employed to provide an ethical framework around embryo research would have to be modified.^153^ We are also cognizant that some communities, in various parts of the world, may be more sensitive to these concerns than others.

Living systems' research that replicates essential components of existing life forms may also raise questions regarding whether there should be limits to scientific efforts that “re-create” natural systems. Foundational to this discussion is the complexity of and current lack of full understanding of the underlying biology and its emergence. Limited knowledge on biological emergence makes it difficult to determine or project what the consequences might be of current research. Indeed, the concept of emergence in complex systems raises the prospect of unforeseen outcomes in the M-CELS that are created, with potentially negative consequences. Efforts to understand and incorporate system repair and healing pathways should also be assessed parallel to efforts to control or limit potential harmful outcomes. Designing “kill-switches” to halt unforeseen harm provides one possible pathway but one that is potentially at odds with efforts to support system self-repair.

A consequence-based analysis would include an assessment of the potential for harm and dual use—in which the technology itself or resulting living systems may have unforeseen, harmful applications with public health and security implications (e.g., bioterrorism). This analysis would also require discussion of possible safeguards that could be explored throughout the research process.

Medical applications of M-CELS, particularly in connection with the creation of non-natural organs (e.g., ones that might synthesize and secrete therapeutic factors for chronic illness) or tissues with enhanced capabilities (e.g., a muscle “patch” containing cells that out-perform natural muscle and thereby enhance athletic performance or an implant that enhances mental acuity) raise new issues not previously considered. At what stage do we limit the use of such performance enhancements? Would they only be accessible to a select few? Numerous scenarios can readily be envisioned which raise important ethical questions for society.

The asserted goal of work in M-CELS is to support the greater good of society, identifying constructive, efficient, new pathways for solving functional, real-world needs. As research in M-CELS advances, acknowledging both “precautionary” and “proactionary” risk management approaches/principles will be essential. The precautionary principle advises that when there is a potential that harm may occur, researchers should use extreme caution, stopping research until the potential for harm can be assessed.^154^ The proactionary principle, on the other hand, views research as positive for society and assumes that research is beneficial unless there is evidence to the contrary.^155^ An intermediate ethical position may provide a reasonable commitment to both accountability and responsibility. Collaboration between researchers would provide a system for checks and balances, with sharing of information between labs, while acknowledging the tension such sharing would have with pathways for preserving research/publication rights. M-CELS researchers, in close consultation with bioethicists, should develop a shared ethics framework and consider creation of a shared governance structure.

It is unclear to what degree researchers and trainees who work on M-CELS are considering or fostering discussion of these questions surrounding “creating” or “re-creating life” or controlling emergent behavior in their daily work. Those involved in research may benefit from an intentional structure for identifying underlying, competing values and ethical principles in their work and providing a forum for discussion, especially for trainees, about how scientific values and personal values or beliefs coincide. The development of such a frame work and an ethics code of conduct for M-CELS research is an imperative task for the research community to develop. Being able to articulate the ethics underlying one's work is so important in communicating within the scientific community, with funders and policy makers and with the public at large.

## XI. CONCLUSIONS AND OUTLOOK

Following on recent advances in understanding single cell behavior^156^ and in developing simple, proof-of-concept biological machines,^108,112^ organoids,^6^ and organ-on-chip technologies,^84^ efforts are underway to develop the scientific and engineering principles that will ultimately enable the development of M-CELSs. The approaches, however, are widely divergent and often lack a sound basis due to the absence of a fundamental understanding of aspects unique to M-CELSs—complexity, the central role of emergence, and intrinsic variability in the starting components—and fail to take advantage of their extraordinary capabilities—self-assembly, growth, self-repair, adaptation, learning, etc.

In this opinion piece, we argue for the need to build on our current knowledge base for the development and design of M-CELS, rethinking much of what we have learned from abiotic engineered systems and to extend the concepts of synthetic biology to multi-cellular systems. A major effort is required to characterize, model, and image the dynamical behavior of M-CELS and consequently establish the design principles needed for their robust manufacture. Here, we have presented some of the challenges that lie ahead in several of the key scientific and engineering disciplines, as well as the potentially transformative benefits that could be derived from this work. Much research is yet needed to understand cell population behavior to a level commensurate with the need to establish sound principles for the development and design of future M-CELS and to also develop an ethical framework for such research. Despite the challenges, however, the potential benefits are enormous. While the scientific barriers are considerable, no less are the social barriers that need to be addressed as we enter into this new era of engineered living systems.
